# Factory Labour

**Published:** 1833-06

**Authors:** 


### REPORTS

### FROM HOSPITALS AND MEDICAL INSTITUTIONS.

### FACTORY LABOUR.

*On Factory Labour.* By John Malyn, Esq. [Read before the Westminster Medical Society.]

In the early part of the season,* I directed your attention to the subject of Factory Labour. In addition to its importance, it had then the attraction of novelty, and, although the members concurred in my views, yet the Society was induced to adopt a proposition, that, as it was a question in which the well-being of many thousands was concerned, it should be submitted to further consideration.

Many circumstances contributed to frustrate my intention of complying immediately with your wish, and, before they had ceased to operate, the Report of the Factories' Labour Regulation Bill Committee was published.

The appearance of that Report seemed to determine the question. No diversity of opinion appeared to exist. Scarcely was there a voice heard on the matter, but it was raised in reproval of a system which smites the innocent and the young, which mars the structure and deforms the mind of youth, which uproots the best interests of man, annihilates his domestic happiness, and severs his socialities.

In the midst of such unanimity of opinion, it appeared to me to

* Vide No. 80 (n.s.), p. 109, of the present volume.

be useless to moot a subject about which there was no dispute, and on which all classes of men had made up their minds.

The case is changed; after all this toil and anxiety to establish the justice of their cause, the benevolent individuals who have laboured for the young are driven back to their proofs. A recent decision of the House of Commons has sent it forth to the world, that the members of that house still doubt whether it is injurious to childhood to be employed from morning until night as factory-people are employed; and they, who frequently complain of the fatigue of their own labours, have actually appointed a commission to ascertain whether a child cannot *stand* more than ten hours a-day in an elevated temperature, and breathing a pernicious atmosphere. Be it therefore our task to examine the reasonableness of their doubt.

In a subject of this nature, it is expedient that the point for which we contend should be clearly stated, and separated from those accidental bearings which, to a properly-constituted mind, are at most but of secondary importance.

It is the more necessary to do this, since those who are opposed to a restriction of the hours of labour have always evaded the real question, and have adverted only to the probable operation of such a measure upon trade; but with arguments of this description we can have no concern. Interests which have been created by, and which are founded on, an abuse, can surely prefer no substantial claim to consideration.

Assuming, then, that this globe was destined for the reception of active and efficient beings, who, though designed for labour, should labour in comfort, and to whose health indeed the exercise should contribute, I take it that the most honest end we can propose to ourselves is, to ascertain generally whether man, in his full formation, is endued with powers to resist the universally-admitted circumstances under which factory-labour is pursued, and particularly whether it can be reconciled with the known laws of the animal economy, that nature shall be enabled to develop the human body, when submitted to its influence during the period of growth.

To resolve these points satisfactorily, it is necessary that we correctly understand each other when we speak of the fitness of the body for labour, and of the capacity of the body for labour. We may then proceed with our inquiry into the effects of moral and physical agents, as they promote, preserve, or depreciate this fitness and this capacity.

The condition in which the organized structure is fitted for labour is that in which the different parts of our complicated machine have attained their growth, and are in harmonious consent each with the whole, not one more than another being predominant in the mind. The mass of matter of which the body is formed is an aggregation of instruments of two kinds; one, by means of which the mind is made manifest, and relation to the rest of the species secured; and the other is to provide for the sustentation of the former. Both these orders are combined and formed into a whole by the termination, at a common centre, of the nervous filaments which pervade all parts, however distant, or however minute.

An identity of sensation, apart from all considerations of matter, is thus produced; and it is only when the attention is purposely directed to the different organs, or when they act in obedience to the will, that the mind is conscious of their existence as parts.

This, and this only, is the state of health, and the condition, in which the body is fitted for labour. Whatever occupations may be pursued, whatever agents the system may be exposed to, so long as this unity of formation is preserved, so long as the subject has it not obtruded on him that he has an arm, or a leg, or a head, or anything else, thus long may that occupation, and those agents, be pronounced salutary, and such as the being was formed to follow and endure. But, whatever may destroy this singleness of sensation, whatever may cause one organ, or one group of organs, to be eternally, or even occasionally, present to the mind, is by that very effect proved to be obnoxious, and contrary to the design of nature.

A state of health, therefore, is that in which we have a consciousness of identity, unincumbered by any impression of the existence of organized instruments. Thus we have an unerring criterion of the "fitness of the body for labour." This state of physical excellence is relative to another condition, and is therefore variable in its degree of perfection; for two given bodies may each be fitted for labour, and at the same time may differ widely in the extent of their respective capacities. Indeed, capacity and fitness go hand in hand, and, by their reciprocal influence, improve each other.

This capacity is dependent on a subtle principle, which we know only by its effects, but which vivifies, and renders available for the purposes of life, organs which, though perfect, would, without it, be passive. It confers activity in proportion to its abundance, and is itself consumed by the action it originates, and that too with a rapidity increasing as the action is uniform and enduring. We stretch forth an arm; it is a simple action, and for a short period may be easily endured, but, if we maintain that arm in such a position somewhat longer, it will so drain the principle of which we are speaking, that languor, indicative of its exhaustion, will quickly supervene; and were we, nevertheless, to push our efforts still further, universal prostration would ensue. The nervous system being the medium of its transmission, we may at once comprehend how it is that the action of a single muscle can produce this general effect.

It is not therefore the amount of action, when properly and universally distributed, that consumes this principle, so much as its localization and continuance. It is not voluntary muscular action alone which effects its exhaustion, but it is action of every kind; for, whether it be of the mind or of the body, of the organs of life or of the organs of relation, the result will be the same.

This principle is regenerated by rest, which rest must be proportioned to its previous consumption. The natural action, which provides for the maintenance of the body, of itself abstracts it so far as to induce periodic sleep, that it may then reaccumulate; and as, in the common labours of life, there is superadded voluntary action, there must be a more speedy exhaustion, and, consequently, a necessity for more frequent rest. Thus the custom of society has divided the day in such a manner that, by refreshment and rest, the patient may reacquire that principle of excitability on which h s efficiency depends. This applies to the repose of the general system; but we must remember that there is a local regeneration of excitability, produced by the temporary quiescence of the employed muscles, by means of which the general exhaustion is more gradual, and is longer deferred.

These considerations teach us—

That the maintenance of one position, or the performance of one act, for a given period, will consume the excitability more than a variety of postures, or diversified employments, will in twice that time;

That rest of longer duration, and of more frequent occurrence, will be necessary for its reaccumulation in the former than in the latter case;

That action of the organs of the mind, and action of the organs of locomotion, cannot proceed as efficiently together, or to the same extent, as either of them separately;

That action of either of these two sets of organs is incompatible with vivid action of the organs of life;

That when the periods of repose do not correspond, in number and duration, to the extent of the exhaustion, an atonic state of body will be produced.

A general observation of natural employments will instruct us that these considerations are kept in view. The motions of the labourer are strikingly varied, and, in a very short period, almost every muscle in the body is, in its turn, stimulated into action. In the midst of this general activity there is, however, individual quiescence sufficient to admit of the recovery of a certain degree of power; and these alternations proceed until the combined exertion shall have gone so far as to have sensibly reduced the excitability. This marks the period for the reception of food, and the repose in which the labourer indulges, (as we see in the fields, and even in the streets,) facilitates its appropriation, and admits of a considerable restoration of his energy. He resumes his labour until the same state is again induced, when it is again similarly surmounted. A few of these periods reduce the powers so far that a longer interval of rest is demanded, and nature has bequeathed it to the whole creation during the hours of night.

The purity of the atmosphere they respire, as well as the quick transmission of the blood to the heart and lungs, by reason of the varied action of the muscles, eminently tends to maintain and increase the integrity of the body. We see, on the other hand, where a life of restricted exertion is pursued, where the activity of the same set of muscles is preserved, not only is the tone of these muscles affected, but that of the whole body considerably diminished, its condition impaired, and its dissolution anticipated. In many trades which the artificial state of society has called into existence, this fact is strongly exemplified. True, it does not strike a casual observer with much force; for, from his youth upwards, he has been familiarized to the sight, and there is nothing unnatural to him in the appearance. Milliners, tailors, shoemakers, and many others of like occupations, will feelingly describe the agony which embitters the early years of their introduction to labour, and the relief, the joy, which they experience from a temporary change of employment. But I hear it alleged, in the common formula of speech, that "use is second nature, and that they become familiarized to the pursuit." They do indeed! but at what expense, is testified by the records of our hospitals, and the experience of our profession. They do indeed! but it is as the overstrung bow preserves its form when its elasticity is lost, that they retain the outward characters of their species after they have lost the resistency both of body and of mind. The evils which beset them lurk beneath the surface, and do not, by their prominency, warn them of their danger. Torpidity succeeds this unnatural excitement, and the sense of suffering ceases. Day glides after day, and they are conscious of no change as time advances; nor can they, but by contrasting distant periods, appreciate the alteration they have sustained. It originated they know neither how nor when; yet this they know, that their life is but a life of endurance, for which the world offers but few charms, and death scarcely any terrors.

Here then we may view some of the lights and shades of a labourer's life. Here we may see the means whereby health is preserved, and labour lightened; and here we may contemplate the awful penalties of an interference with the laws of nature.

We might now proceed to inquire how far the circumstances of factory-labour correspond with the necessities of man. It is essential, however, to notice previously the peculiarities of childhood, since it is to that state our investigation principally refers. These peculiarities will demonstrate, with an irresistible emphasis, that the labour of a factory-child is the labour of death, and that misery is its inevitable portion.

A momentary glance at the common term of life suggests a division of it into three stages. In the first, the instruments of the body are developed, augmented, and gradually matured; in the second, the organization is complete, and its fitness for labour established; the third is the period of decline.

In the first, the plastic or formative period, we discover no provision for laborious pursuits, but, on the contrary, we find a condition of parts which strictly forbids them. Everything is here incomplete: the bones, designed for strength to the limbs, are not consolidated; the muscles, by which they are moved, have not acquired their power. It is the period of nature's exertion, in which she is vigorously employed in building up the human fabric, and in manufacturing implements for the future use of the subject. Here there is much work to be done, many changes to take place, and much matter to be prepared and deposited in the body, as well for its sustentation as for its growth. The organs employed in the preparation and transmission of this material are necessarily in vivid action, an action which, if it occurred in the second stage of life, would be termed inflammatory. To answer such excessive demands, repeated exhibition of food is requisite, and means to secure its perfect assimilation required. These means are, frequent rest, (that the principle which supports functional excitement, and which this inordinate activity destroys, may be regenerated,) good air, and exercise, varied and apportioned to the age of the child.

This feverish state of growth induces a great sensibility of the surface of the body to external impressions, which requires that it should be strictly guarded from vicissitudes of temperature and of atmosphere, that the balance of action be not disturbed.

As development proceeds, and tone is established, so the degree of excitement is reduced, until, at the expiration of about a fourth of the natural term of existence, the powers of life become restricted to the simple preservation of the body, and the plastic merges into the second, or active period.

Now it is that we discover a condition of body peculiarly fitted for labour. The interior functions are tranquil, the object of their previous intensity having been accomplished; and they therefore do not make anything like the call on the principle of excitability which they did before. A great supply is consequently provided for the organs of relation and these organs; those instruments, or tools, wherewith the being is to work, are finished, and adapted to the end for which they were formed. As nature points out, in the first period, that she does not require the subject to make use of imperfect instruments, any more than we expect a carpenter to work with tools before the materials of which they are constructed have been modelled, completed, and formed into an efficient whole, so, as we look for industry to this carpenter when his instruments are perfected, does she design that all the powers of man shall be put forth in this, the second, or active, period of existence.

Enduring for about twice the previous term, this is succeeded by the third, or period of decline.

The duties of the former stage fulfilled, preparations for departure commence. The powers of life are in gradual recession; the organization loses its resistency; function after function is impaired, and often lost; and the principle of excitability becomes less and less abundant, until, by its entire abstraction, the phenomena of life can no longer manifest themselves, and the whole is terminated by the condition called death.

In noticing these stages of existence, we cannot fail to be struck with the remarkable provision which has been made for each. Great internal action, to supply the demands of the first; strength and vigour for the activity of the second, and an adaptation of power to the diminished energy of the third; hence we may, by an inspection of the organization, at all times infer the duties of every age, and ascertain the degree of labour which nature requires, or can tolerate; inasmuch as the absence or imperfection of organs required for the performance of a given act, is ample evidence that she did not intend such act to be attempted by the subject at that particular period. To exact, then, from the first period of life the labours of the second, would be as unreasonable as to expect that infants should masticate before dentition, or procreate their species before sexual development.

If we turn a deaf ear to the appeal of reason, and shut our eyes to these indications, what deplorable consequences must we not anticipate, if we interfere with nature while she is so unremittingly engaged? If the intervals of rest do not correspond, in number and extent, to the internal commotion, the efficiency of the organs of life must be depreciated in proportion to the privation. If there be superadded external exertion, the excitability will be more rapidly and more effectually exhausted, the functions of life will be enfeebled, the structure vitiated, and the plastic period will be cut short, and will pass into the period of decline, without the intervention of the second.

Having glanced at the different conditions of the human body, at the peculiarities of childhood, and at the circumstances which are essential to its welfare, it remains but to inquire whether those which obtain in our factories correspond with its exigencies.

Some of the statements which have been rendered on this point are scarcely credible. Postures the most constrained and irksome to be borne, hours of exertion extended far beyond what charity can contemplate, an atmosphere palpable to the senses from its grossness, humidity saturating the clothing, an abridgment, nay, denial of periodic refreshment, and an inhuman violation of the laws of nature, in filching from the operatives that nocturnal repose which is not denied to the very beasts of the field, are recorded in burning letters of shame, as being the circumstances which, in many instances, childhood has been compelled to endure.

Such atrocities however are not, they cannot be, universal; but I grievously err, if the mildest form of factory labour, pursued under the most humane of masters, is, in any one of its circumstances, adapted to the plastic state of youth.

If we select the best examples for our consideration, we shall find that in all there is an elevated temperature, in all there is uniformity of labour, in all there is a polluted atmosphere, in all there is an undue limitation of the periods of repose.

At an early hour a child is roused to its labour, even in the winter months, and, in a state of excitement, it is exposed, for a quarter of an hour or more, to the sleet, or the rain, or the frost, but certainly to the cold. In a temperature of sixty or seventy, or eighty, or ninety degrees, for fourteen hours, (saving half an hour for the morning, an hour for the midday, and half an hour for the evening meals,) it must stand on its legs, scarcely ever moving, and occupied, during the major part of the time, in watching a number of threads in rapid progress through the machinery, that, when they break, it may seize, and combine their disunited ends. This it does in an atmosphere from which the fresh air is excluded, which has been breathed over and over again by the persons employed, which is loaded with the exhalations from reeking bodies, and which, in many instances, is charged with dust and flue. Before its duties are completed, oil or gas lights are brought into requisition; and these consume still further the vital properties of the atmosphere, and produce a greater heat. External temperature is at its lowest point; humidity, possibly, is more established; noxious exhalations, through which none but the healthy and the strong can pass unscathed, are issuing from below and descending from above, when the moment of its emancipation arrives. Through such circumstances it is that, scantily clothed, the exhausted child must pass, after leaving a tropical temperature, to seek a few hours of repose. And morning after morning, day after day, and night after night, must it run the gantlet through these deteriorating agents. And whither does it go? To a denselypeopled colony, where the houses are huddled together, and where every room, from the cellar to the garret, has many inmates. Within, these houses are all wretchedness, and destitute of a single vestige of domestic comfort; without, the state of things is more revolting. The streets, seldom paved, and, when paved, never cleansed, are the depositaries of many abominations; in them, animal and vegetable matter, in every stage of decomposition, mingles with the accumulated filth, rendering them at once offensive and nearly impassable.

Had it not been that those who are considered wise in their generation have, by their vote, shewn that they think differently, we might, in our simplicity, have fearlessly declared that it was impossible to doubt whether the growing frame could, without certain danger, thus bare its breast to the storm, or with impunity thus defy the laws of its Creator, and mock his power.

There is not in this description of labour a single circumstance which is necessary for the period of growth; there is not good air, there is not varied exercise, there are not proper intervals of rest. But there are all, and more than all, those circumstances which have been pointed out as destroying the excitability of the system, and as impeding, and even altering, the functions of life.

The labour itself, at the early age these children are consigned to it, is more than sufficient to neutralize the efforts of nature, and to prevent their attaining a robust meridian. The vital columns of flesh which tug and strain for twelve hours a-day to maintain the head on the trunk, and the trunk on the pelvis, to balance these on the thighs, to counteract the flexure of the knees, and, finally, to preserve the whole on the arches of the feet, must quiver with agony whilst they retain any healthy force.

When we carry our thoughts still further, and reflect on the incompleteness of the bones, do we not find in *their* state a direct prohibition of such employment? And must it not create a feeling of astonishment, that, numerous as are the deformities which occur, they are not yet more numerous?

What but a weak and diseased body can be formed from the product of jaded organs? What but a feeble and vitiated mind from such an unnatural consumption of energy?

The sensibility of surface, induced by the operation of heat, will subject the system, when exposed to low temperature or humidity, to all the consequences of disturbed circulation. In those who have been recently introduced to the employment, there will be great febrile excitement and local congestion. It will be different with those who have seemingly withstood its effects for sometime. They have become enfeebled, and here such tonic changes will be rare; but they yet yield to its influence, although the effects are not immediately developed. Insidious though it be, we can be in no doubt as to what really takes place, and we can readily determine that the action and collapse which attend such excitement, and such exposures, must materially affect the organs of life, and, in particular, derange that important viscus, the liver, disqualifying it for the performance of its functions, and contributing thereby, with the diminished excitability already alluded to, to produce a general torpidity of structure.

It is natural to suppose that the continued respiration of an atmosphere which is loaded with foreign matter will diminish the capacity of the lungs, and stimulate them to local action; and such, we hear on all hands, is the fact in those departments where much dust prevails. It has been particularly adverted to by Mr. Thackrah, of Leeds, who, by means of a pulmometer which he has devised, has ascertained that the lungs of those who pursue dusty occupations will not admit nearly so much air as the lungs of those who are otherwise employed.

Bearing this in mind, and reflecting on the change in the constitution of the atmosphere effected by the accumulation of so great a number of persons, the decomposition of the oil, the attrition of the machinery, and the gas-lights at night, it will be apparent that the arterialization of the blood cannot be accomplished. The passive nature of the employment will also favour a languid state of circulation, by which the transit of the blood through the lungs will be less frequent than when the actions of all the muscles contribute to its propulsion. Another means, by which the energy of the body is effected, here presents itself; for, owing to this imperfect decarbonization, that which was designed to be a refreshing stream of life, carries with it a sedative principle which destroys the tone of every part within its circle.

All these causes, though differing in some of their effects, unite to impair that function which provides for the atomic changes which are constantly taking place, and an impoverished state of body ensues.

To feel surprised, then, at seeing a weak and stunted race, mere spectres of men and women, would be to acknowledge our ignorance of the operations of nature in the development of the human body; and to express astonishment at the universality of scrofula, and other affections of debility, would be to confess that we know nothing of the cause of disease.

But most of these came healthy into the world, and it has been the force of circumstances which has marred them. What will their children be? The weakness and morbid tendencies of such bodies will, to a greater or less extent, be impressed on their offspring, and beings of an inferior structure will start into existence to perform their part in life. These, treading the same steps their sires had trod before them, and exposed to the same agents to which they had been exposed, will, with their diminished capacity of resistance, more readily yield to the influence of these causes. In their turn they may, perchance, become parents, but it will be of children who will have still further declined from the standard of health, and who will be born in misery, and cradled in vice. Can we advance another step, and suppose this generation capable of continuing its species? I think not; but, granting the possibility, the produce would be a race the very incarnation of deformity and disease, unfit for any duty, and made as it were in mockery of man.

Hence we might with certainty anticipate that, if the factories were not to be supplied from other sources than their own population, if the sister-kingdom and the agricultural districts were to be closed against them, not a century would elapse before the class would be exterminated, and the mills and the machinery left, in the midst of desolation, nothing but monuments of human ingenuity, and of want of human foresight.

There is another consideration, independent of the effect of this system on the organization, of deep interest to society at large; and, though it is not essentially connected with a medical view of the subject, it may not be amiss to notice it briefly. An attention to what has been previously advanced will shew that the circumstances of factory-labour are directly opposed to the cultivation of morals. Indeed, the mere aggregation of the two sexes, to endure together any tiresome employment, is unfavorable to such a purpose; and still more unfavorable is an occupation pursued in an elevated temperature, and in a state of comparative nudity. When the young are made constant witnesses of improper behaviour and indelicate language in adults, of whom they are unavoidably imitators, the feeble barriers of virtue are soon broken down, and they initiated into vice.

But, however powerful example may be, there will be found, in the nature of their employment itself, causes which tend to demoralize them. The excessive heat by which they are surrounded effects a premature development of the animal propensities, before the mind can attain sufficient strength to coerce these passions. Even in situations where facilities are afforded for the cultivation of the mind, individuals of a superior class to these poor creatures give way to licentiousness, to overcome the torpidity which extreme heat induces, and the voluptuousness of the inhabitants of warm climates may plead a strong apology for the laxity of factory-labourers.

Again, the physical effects which have been enounced as influencing the functions of the organs of life and of locomotion, extend themselves with equal force to the mind; and the organs by which it acts are laid as prostrate as the instruments of the body. Deprived of that presiding power which should regulate and balance all their proceedings, they are incapable of thinking before they act. They seek excitement of any kind, because they thereby obtain a temporary exemption from suffering. They flog the organs of their mind into something like activity, by the indulgence of ideas of a stimulating character, and by indecent gestures and conversation, as they do those of the body by drinking ardent liquors. They have not, they cannot have, from the condition of their organization, any principles to check such practices, or to make them sensible of their error. And what is the consequence? That close on the heels of this excitement follows greater torpidity, which, similarly overcome, is yet more and more increased until the system ceases to be influenced by the stimulus.

To counteract the demoralizing tendency of factory-labour, it has been customary to request, and sometimes to enforce, the attendance of the children at school on Sunday.

I would not willingly impugn the efficacy of these establishments under common circumstances, but I would suggest to their well-meaning yet assuredly mistaken supporters that they are themselves violating the laws which they wish to be observed, by working on the seventh day a fagged and wearied child, by depriving him of the only opportunity he has to regain a portion of his energy, and by blotting out, though meant in kindness, the bright spots that are scattered, few and far between, in the black catalogue of his days.

Such is the view I take of this important question, and such the reasons which lead me to conclude that

*Factory labour is, in all its circumstances, unfavorable to the maintenance of health, even in the middle period of life:* and that

*During the period of growth it is directly opposed to the development of mind, and of body, and of morals; is certain to introduce disease, and will considerably shorten life.*

Should these conclusions be admitted, though towns be razed and trade annihilated, the system ought to be instantly and effectively corrected. It devolves on our profession (of which it is the proud distinction to alleviate human suffering) to chase away the doubt of the destructiveness of factory-labour, which is implied in the recent appointment of a commission, as uncalled for as it will be inoperative; a commission which cannot alter or subvert the laws of nature, but which will meet deceit at every step, and falsehood at every turning. It is for us, at all times and in all societies, perseveringly to assert and distinctly to prove that factory-labour is nothing but a species of *refined and protracted murder.*

Man may thus be made sensible that there is a duty of a higher order, and of a nobler character, than that of amassing wealth and building palaces—the duty of observing, in spirit and in truth, that social, that moral, that divine law, "do unto others as ye would they should do unto you."

